# Supplementary material for: Deletion of wfs1 Impairs Oligodendrocyte Precursor Cells Dorsal Distribution and Myelination Through the wfs1-hmgcs1 Axis in Zebrafish
Source: Biology (Basel). 2026 Aug 16;15(16):1402. doi: 10.3390/biology15161402 (PMC13510151; doi:10.3390/biology15161402)
Supplement: Supplementary file 1 [file biology-15-01402-s001.zip › File S1-Transcriptome sequencing data and differentially expressed genes (DEGs) in wfs1-- zebrafish at 5 dpf/2.KEGG/wfs1KOvsWT/DOWN/wfs1KOvsWT.down_KEGGenrich.html]

Pathway Enrichment


# The most enriched pathway terms

Statistic method: hypergeometric test

FDR correction method: Benjamini and Hochberg

| Term | Description | Sample number | Background number | P-value | Corrected P-value | Gene\_id | Gene\_name | KEGG\_ID |
| --- | --- | --- | --- | --- | --- | --- | --- | --- |
| dre00053 | Ascorbate and aldarate metabolism | 9/389 | 66/8379 | 0.00322933166691862 | 0.424759718364805 | 100005587\_1 100536795 403070 406731 100415796 570913\_1 445326 767756 100006238\_1 | aldh9a1a.1 wu:fb14d09 rgn ugt1ab ugt5b5 ugt5g1 akr1a1a ugt5a2 aldh9a1a.2 | dre:100005587 dre:100536795 dre:403070 dre:406731 dre:678597 dre:570913 dre:445326 dre:767756 dre:100006238 |
| dre00590 | Arachidonic acid metabolism | 9/389 | 72/8379 | 0.00581862627896993 | 0.424759718364805 | 406449 767759 406370\_1 494108\_1 797309 566996\_1 399485 767688 100148818 | ptges3a zgc:153704 pla2g6 ptgr1 cyp2j20 si:dkey-11k2.7 cyp2p10 zgc:154040 si:ch73-55i23.1 | dre:406449 dre:767759 dre:406370 dre:494108 dre:797309 dre:566996 dre:399485 dre:767688 dre:100148818 |
| dre04150 | mTOR signaling pathway | 20/389 | 251/8379 | 0.012741572815707 | 0.610802909062199 | 324140 336619 436718\_1 100333504\_1 359840\_1 100000252\_1 573492 792689\_1 79380\_1 245702\_1 563708 556087\_1 555584 58072 30369\_1 791993 790938 30127\_1 797250 497384\_1 | sgk1 eif4bb rhocb lamtor5 atp6v1b2 pik3r3b rragca mlst8 eif4ea igf1rb skp2 wnt2bb prr5a fzd8b fzd7a wnt4b zgc:158423 wnt2 slc7a5 prkca | dre:324140 dre:336619 dre:436718 dre:100333504 dre:359840 dre:100000252 dre:573492 dre:792689 dre:79380 dre:245702 dre:563708 dre:556087 dre:555584 dre:58072 dre:30369 dre:791993 dre:790938 dre:30127 dre:797250 dre:497384 |
| dre00591 | Linoleic acid metabolism | 5/389 | 33/8379 | 0.0170594147185192 | 0.610802909062199 | 406370\_1 797309 399485 767688 100148818 | pla2g6 cyp2j20 cyp2p10 zgc:154040 si:ch73-55i23.1 | dre:406370 dre:797309 dre:399485 dre:767688 dre:100148818 |
| dre00010 | Glycolysis / Gluconeogenesis | 10/389 | 103/8379 | 0.0209179078445959 | 0.610802909062199 | 560753 100005587\_1 100536795 568001\_2 406339\_1 561416 393427 445326 436646 100006238\_1 | tpi1b aldh9a1a.1 wu:fb14d09 pfkmb hk2 pfkpb zgc:63568 akr1a1a galm aldh9a1a.2 | dre:560753 dre:100005587 dre:100536795 dre:568001 dre:406339 dre:561416 dre:393427 dre:445326 dre:436646 dre:100006238 |
| dre01230 | Biosynthesis of amino acids | 10/389 | 109/8379 | 0.0296567398628463 | 0.721647336662593 | 560753 568001\_2 322670\_1 550579 550524\_1 407673 561416 406688 334195 678634 | tpi1b pfkmb aco2 idh3g cbsa rpia pfkpb got2a mat1a mat2al | dre:560753 dre:568001 dre:322670 dre:550579 dre:550524 dre:407673 dre:561416 dre:406688 dre:334195 dre:678634 |
| dre00380 | Tryptophan metabolism | 7/389 | 67/8379 | 0.0349675371497214 | 0.729322917694189 | 100005587\_1 100536795 368262 393860\_1 447801 571201 100006238\_1 | aldh9a1a.1 wu:fb14d09 dlst gcdha aldh8a1 si:ch73-97h19.2 aldh9a1a.2 | dre:100005587 dre:100536795 dre:368262 dre:393860 dre:447801 dre:571201 dre:100006238 |
| dre03410 | Base excision repair | 6/389 | 58/8379 | 0.0511536576018448 | 0.75584048389129 | 321622 393774\_2 556995\_2 553405\_1 558198\_1 796446\_1 | hmgb1a pole3 lig1 pole si:ch211-141o9.10 adprhl2 | dre:110438871 dre:393774 dre:556995 dre:553405 dre:558198 dre:796446 |
| dre00270 | Cysteine and methionine metabolism | 7/389 | 73/8379 | 0.0518971367517084 | 0.75584048389129 | 550524\_1 571201 791167 431773 406688 334195 678634 | cbsa si:ch73-97h19.2 zgc:158222 enoph1 got2a mat1a mat2al | dre:550524 dre:571201 dre:791167 dre:431773 dre:406688 dre:334195 dre:678634 |
| dre00860 | Porphyrin metabolism | 7/389 | 74/8379 | 0.0551436264736277 | 0.75584048389129 | 100002875\_1 406731 100415796 570913\_1 100329531\_1 64607\_1 767756 | hmox2a ugt1ab ugt5b5 ugt5g1 hmox2b alas2 ugt5a2 | dre:100002875 dre:406731 dre:678597 dre:570913 dre:100329531 dre:64607 dre:767756 |
| dre01200 | Carbon metabolism | 13/389 | 173/8379 | 0.0587855776694743 | 0.75584048389129 | 560753 568001\_2 322670\_1 550579 368262 407673 541329\_1 403070 792824\_1 406339\_1 561416 406688 393427 | tpi1b pfkmb aco2 idh3g dlst rpia gcsha rgn pgp hk2 pfkpb got2a zgc:63568 | dre:560753 dre:568001 dre:322670 dre:550579 dre:368262 dre:407673 dre:541329 dre:403070 dre:792824 dre:406339 dre:561416 dre:406688 dre:393427 |
| dre00983 | Drug metabolism - other enzymes | 10/389 | 124/8379 | 0.0621238753883252 | 0.75584048389129 | 492517\_1 406731 100005763\_1 437016\_1 794381 100415796 570913\_1 406703 103910413 767756 | zgc:103586 ugt1ab uck2a tk2 si:dkey-78a14.4 ugt5b5 ugt5g1 gsta.1 tymp ugt5a2 | dre:492517 dre:406731 dre:100005763 dre:437016 dre:794381 dre:678597 dre:570913 dre:406703 dre:103910413 dre:767756 |
| dre04810 | Regulation of actin cytoskeleton | 25/389 | 404/8379 | 0.0858626262218667 | 0.759499660951657 | 57934 286739\_1 30429\_2 793158 436718\_1 566764 100000252\_1 557935 386856\_1 799354\_1 100005049 novel.946 100331581\_1 569566 571431 560564\_2 100536705\_2 553493 323285\_1 378480 393611 445305\_1 767646\_1 797491\_1 792699 | actb1 msna mylpfa cdc42l rhocb scin pik3r3b zmp:0000001082 pdgfra cxcl12b fgf1b - si:dkey-44g17.6 LOC569566 LOC571431 pdgfc itgae.2 arhgef7a scinla pdgfaa c6 arpc4l abi2a itgb7 rac1l | dre:57934 dre:286739 dre:30429 dre:793158 dre:436718 dre:566764 dre:100000252 dre:557935 dre:386856 dre:799354 dre:100005049 dre:386856 dre:100331581 dre:569566 dre:571431 dre:560564 dre:100536705 dre:553493 dre:323285 dre:103909601 dre:393611 dre:445305 dre:767646 dre:797491 dre:792699 |
| dre03015 | mRNA surveillance pathway | 9/389 | 118/8379 | 0.0972389035757666 | 0.759499660951657 | 386630 324267 393693 100005893\_1 492341\_1 394058\_1 563172 562282\_1 559392 | tardbpl hbs1l sap18 magoh pcf11 fus etf1a ppp2r5b ppp2r2cb | dre:386630 dre:324267 dre:393693 dre:100005893 dre:492341 dre:394058 dre:563172 dre:562282 dre:559392 |
| dre00980 | Metabolism of xenobiotics by cytochrome P450 | 7/389 | 85/8379 | 0.0990888314627836 | 0.759499660951657 | 406731 393293 100415796 570913\_1 406703 393427 767756 | ugt1ab hsd11b1la ugt5b5 ugt5g1 gsta.1 zgc:63568 ugt5a2 | dre:406731 dre:100006793 dre:678597 dre:570913 dre:406703 dre:393427 dre:767756 |
| dre00051 | Fructose and mannose metabolism | 5/389 | 54/8379 | 0.104278804344388 | 0.759499660951657 | 560753 568001\_2 406339\_1 561416 100007686 | tpi1b pfkmb hk2 pfkpb LOC100007686 | dre:560753 dre:568001 dre:406339 dre:561416 dre:100007686 |
| dre00920 | Sulfur metabolism | 2/389 | 12/8379 | 0.104372507951521 | 0.759499660951657 | 777719 566675 | papss2a suox | dre:777719 dre:566675 |
| dre00260 | Glycine, serine and threonine metabolism | 5/389 | 56/8379 | 0.117101195075564 | 0.759499660951657 | 394103\_1 541429\_1 550524\_1 541329\_1 64607\_1 | sardh aoc2 cbsa gcsha alas2 | dre:394103 dre:541429 dre:550524 dre:541329 dre:64607 |
| dre00592 | alpha-Linolenic acid metabolism | 3/389 | 26/8379 | 0.117556970664343 | 0.759499660951657 | 406370\_1 767688 100148818 | pla2g6 zgc:154040 si:ch73-55i23.1 | dre:406370 dre:767688 dre:100148818 |
| dre00900 | Terpenoid backbone biosynthesis | 3/389 | 26/8379 | 0.117556970664343 | 0.759499660951657 | 334380 559054 100003383\_1 | zmpste24 hmgcra pmvk | dre:334380 dre:559054 dre:100003383 |
| dre00410 | beta-Alanine metabolism | 4/389 | 41/8379 | 0.120864366835746 | 0.759499660951657 | 100005587\_1 541429\_1 100536795 100006238\_1 | aldh9a1a.1 aoc2 wu:fb14d09 aldh9a1a.2 | dre:100005587 dre:541429 dre:100536795 dre:100006238 |
| dre04217 | Necroptosis | 14/389 | 215/8379 | 0.126083095089531 | 0.759499660951657 | 336426\_1 559768 321622 373107\_1 337413 550542\_1 558816 100126128 564295\_2 368481 100148818 100149436\_1 561021\_1 403131 | chmp1b zgc:173593 hmgb1a birc2 stat6 chmp4c LOC558816 zgc:173594 traf2a stat1b si:ch73-55i23.1 si:ch211-189a15.5 si:dkey-103e21.5 tlr4ba | dre:336426 dre:559768 dre:110438871 dre:373107 dre:337413 dre:550542 dre:558816 dre:100126128 dre:564295 dre:368481 dre:100148818 dre:100149436 dre:561021 dre:403131 |
| dre04120 | Ubiquitin mediated proteolysis | 13/389 | 199/8379 | 0.134449035461692 | 0.759499660951657 | 550134 564149 373107\_1 692325\_1 323951 323883 327215\_2 100136859 100001302 447935\_1 563708 393223\_1 30100 | ubb pias2 birc2 flj11011l klhl13 cul1a cul5a fbxw8 uba7 tceb1a skp2 fzr1a ube2kb | dre:550134 dre:564149 dre:373107 dre:692325 dre:323951 dre:323883 dre:327215 dre:100136859 dre:100001302 dre:447935 dre:563708 dre:393223 dre:30100 |
| dre00052 | Galactose metabolism | 4/389 | 43/8379 | 0.137340921727712 | 0.759499660951657 | 568001\_2 406339\_1 561416 436646 | pfkmb hk2 pfkpb galm | dre:568001 dre:406339 dre:561416 dre:436646 |
| dre00511 | Other glycan degradation | 3/389 | 28/8379 | 0.138644177159788 | 0.759499660951657 | 445250 445250\_1 100000684\_1 | neu3.2 neu3.2 neu3.5 | dre:445250 dre:445250 dre:100000684 |
| dre00030 | Pentose phosphate pathway | 4/389 | 45/8379 | 0.154663805265399 | 0.759499660951657 | 568001\_2 407673 403070 561416 | pfkmb rpia rgn pfkpb | dre:568001 dre:407673 dre:403070 dre:561416 |
| dre04621 | NOD-like receptor signaling pathway | 13/389 | 205/8379 | 0.157306572478697 | 0.759499660951657 | 100000242 436718\_1 373107\_1 557239 436734\_1 564295\_2 323944 100334992 368481 100002946 561021\_1 403131 100149641 | zgc:92606 rhocb birc2 antxr2a txn traf2a si:ch1073-155h21.1 unm\_sa911 stat1b cxcl8a si:dkey-103e21.5 tlr4ba LOC100149641 | dre:100000242 dre:436718 dre:373107 dre:557239 dre:436734 dre:564295 dre:323944 dre:100334992 dre:368481 dre:100002946 dre:561021 dre:403131 dre:100149641 |
| dre00040 | Pentose and glucuronate interconversions | 5/389 | 62/8379 | 0.159666539791025 | 0.759499660951657 | 406731 100415796 570913\_1 445326 767756 | ugt1ab ugt5b5 ugt5g1 akr1a1a ugt5a2 | dre:406731 dre:678597 dre:570913 dre:445326 dre:767756 |
| dre00340 | Histidine metabolism | 3/389 | 30/8379 | 0.160893214637657 | 0.759499660951657 | 100005587\_1 100536795 100006238\_1 | aldh9a1a.1 wu:fb14d09 aldh9a1a.2 | dre:100005587 dre:100536795 dre:100006238 |
| dre01210 | 2-Oxocarboxylic acid metabolism | 3/389 | 30/8379 | 0.160893214637657 | 0.759499660951657 | 322670\_1 550579 406688 | aco2 idh3g got2a | dre:322670 dre:550579 dre:406688 |
| dre03030 | DNA replication | 4/389 | 48/8379 | 0.182064024801239 | 0.759499660951657 | 393774\_2 393241\_2 556995\_2 553405\_1 | pole3 rnaseh2b lig1 pole | dre:393774 dre:393241 dre:556995 dre:553405 |
| dre00071 | Fatty acid degradation | 5/389 | 65/8379 | 0.183003443663265 | 0.759499660951657 | 100005587\_1 100536795 393860\_1 393427 100006238\_1 | aldh9a1a.1 wu:fb14d09 gcdha zgc:63568 aldh9a1a.2 | dre:100005587 dre:100536795 dre:393860 dre:393427 dre:100006238 |
| dre04115 | p53 signaling pathway | 7/389 | 101/8379 | 0.188467444312035 | 0.759499660951657 | 140621 431763\_1 436699 140621\_1 796473\_1 100320152 561021\_1 | casp3a gadd45ab zgc:92658 casp3a tp53i3 steap3 si:dkey-103e21.5 | dre:140621 dre:431763 dre:436699 dre:140621 dre:796473 dre:100320152 dre:561021 |
| dre04210 | Apoptosis | 14/389 | 232/8379 | 0.190321962149634 | 0.759499660951657 | 57934 140621 373107\_1 431763\_1 791531\_1 100000252\_1 552927\_1 564295\_2 140621\_1 58100\_1 559564 561021\_1 246726 558237\_1 | actb1 casp3a birc2 gadd45ab casp6l1 pik3r3b casp6 traf2a casp3a badb eif2ak3 si:dkey-103e21.5 birc5b dab2ipb | dre:57934 dre:140621 dre:373107 dre:431763 dre:791531 dre:100000252 dre:552927 dre:564295 dre:140621 dre:58100 dre:559564 dre:561021 dre:246726 dre:558237 |
| dre00310 | Lysine degradation | 7/389 | 102/8379 | 0.194919754431492 | 0.759499660951657 | 100005587\_1 100536795 368262 393860\_1 556535\_1 100003615\_1 100006238\_1 | aldh9a1a.1 wu:fb14d09 dlst gcdha setd1a prdm2a aldh9a1a.2 | dre:100005587 dre:100536795 dre:368262 dre:393860 dre:556535 dre:100003615 dre:100006238 |
| dre00350 | Tyrosine metabolism | 4/389 | 50/8379 | 0.201156419642936 | 0.759499660951657 | 541429\_1 406688 393427 569363 | aoc2 got2a zgc:63568 tpo | dre:541429 dre:406688 dre:393427 dre:569363 |
| dre00982 | Drug metabolism - cytochrome P450 | 6/389 | 85/8379 | 0.201703246027548 | 0.759499660951657 | 406731 100415796 570913\_1 406703 393427 767756 | ugt1ab ugt5b5 ugt5g1 gsta.1 zgc:63568 ugt5a2 | dre:406731 dre:678597 dre:570913 dre:406703 dre:393427 dre:767756 |
| dre00620 | Pyruvate metabolism | 5/389 | 68/8379 | 0.207505177880682 | 0.759499660951657 | 100005587\_1 100536795 393427 445326 100006238\_1 | aldh9a1a.1 wu:fb14d09 zgc:63568 akr1a1a aldh9a1a.2 | dre:100005587 dre:100536795 dre:393427 dre:445326 dre:100006238 |
| dre00830 | Retinol metabolism | 7/389 | 104/8379 | 0.208082098890865 | 0.759499660951657 | 406731 100415796 553239 570913\_1 393427 407663 767756 | ugt1ab ugt5b5 lrata ugt5g1 zgc:63568 si:ch211-107o10.3 ugt5a2 | dre:406731 dre:678597 dre:553239 dre:570913 dre:393427 dre:407663 dre:767756 |
| dre04370 | VEGF signaling pathway | 7/389 | 104/8379 | 0.208082098890865 | 0.759499660951657 | 793158 565241\_2 100000252\_1 58100\_1 100148818 497384\_1 792699 | cdc42l ppp3ccb pik3r3b badb si:ch73-55i23.1 prkca rac1l | dre:793158 dre:565241 dre:100000252 dre:58100 dre:100148818 dre:497384 dre:792699 |
| dre04310 | Wnt signaling pathway | 16/389 | 278/8379 | 0.220559862463255 | 0.773146297430953 | 436718\_1 565241\_2 678649 323883 556087\_1 58072 30369\_1 791993 402843\_1 30127\_1 30197 100006858\_1 798564 326875\_1 497384\_1 792699 | rhocb ppp3ccb zgc:136929 cul1a wnt2bb fzd8b fzd7a wnt4b sfrp1a wnt2 dkk1b csnk1e sfrp1b cby1 prkca rac1l | dre:436718 dre:565241 dre:678649 dre:323883 dre:556087 dre:58072 dre:30369 dre:791993 dre:402843 dre:30127 dre:30197 dre:100006858 dre:798564 dre:326875 dre:497384 dre:792699 |
| dre05168 | Herpes simplex virus 1 infection | 13/389 | 220/8379 | 0.222411948576027 | 0.773146297430953 | 140621 373107\_1 100000252\_1 565338\_1 30400 564295\_2 140621\_1 406288\_1 58100\_1 559564 368481 30163 561021\_1 | casp3a birc2 pik3r3b eif2ak1 b2m traf2a casp3a srsf1a badb eif2ak3 stat1b tapbp.1 si:dkey-103e21.5 | dre:140621 dre:373107 dre:100000252 dre:565338 dre:30400 dre:564295 dre:140621 dre:406288 dre:58100 dre:559564 dre:368481 dre:30163 dre:561021 |
| dre03018 | RNA degradation | 7/389 | 108/8379 | 0.235354827316418 | 0.799111739260396 | 568001\_2 553665 324574 448949 566017 565659 561416 | pfkmb cnot6l wu:fc33b09 cnot1 lsm8 edc4 pfkpb | dre:568001 dre:553665 dre:324574 dre:448949 dre:566017 dre:565659 dre:561416 |
| dre04672 | Intestinal immune network for IgA production | 3/389 | 37/8379 | 0.245415819329504 | 0.807569167780319 | 799354\_1 100006078\_1 797491\_1 | cxcl12b ccr9b itgb7 | dre:799354 dre:100006078 dre:797491 |
| dre04216 | Ferroptosis | 5/389 | 73/8379 | 0.250479099644704 | 0.807569167780319 | 559768 558816 100126128 790938 100320152 | zgc:173593 LOC558816 zgc:173594 zgc:158423 steap3 | dre:559768 dre:558816 dre:100126128 dre:790938 dre:100320152 |
| dre05132 | Salmonella infection | 22/389 | 406/8379 | 0.254439600807498 | 0.807569167780319 | 386590 57934 30429\_2 793158 140621 436718\_1 373107\_1 406811\_1 798985 563023 436734\_1 562372 564295\_2 140621\_1 571356\_1 novel.848 100002946 445305\_1 561021\_1 403131 792699 799806\_2 | hsp90b1 actb1 mylpfa cdc42l casp3a rhocb birc2 zgc:55461 vps16 flnb txn snx18a traf2a casp3a pik3c2a - cxcl8a arpc4l si:dkey-103e21.5 tlr4ba rac1l anxa2b | dre:386590 dre:57934 dre:30429 dre:793158 dre:140621 dre:436718 dre:373107 dre:336681 dre:798985 dre:563023 dre:436734 dre:562372 dre:564295 dre:140621 dre:571356 dre:393993 dre:100002946 dre:445305 dre:561021 dre:403131 dre:792699 dre:799806 |
| dre00360 | Phenylalanine metabolism | 2/389 | 22/8379 | 0.272208104301111 | 0.816076257715589 | 541429\_1 406688 | aoc2 got2a | dre:541429 dre:406688 |
| dre00450 | Selenocompound metabolism | 2/389 | 22/8379 | 0.272208104301111 | 0.816076257715589 | 777719 571201 | papss2a si:ch73-97h19.2 | dre:777719 dre:571201 |
| dre04260 | Cardiac muscle contraction | 10/389 | 173/8379 | 0.283029161794841 | 0.816076257715589 | 393793\_1 64614 447942\_1 100037358\_1 559015 678560\_1 353247 556638\_1 114457 436768 | uqcrc1 atp1a1a.3 cox6a2 zgc:163073 cacnb3b cacnb4b tnnc1a slc9a6b atp1b2b uqcrq | dre:393793 dre:64614 dre:447942 dre:100037358 dre:559015 dre:678560 dre:353247 dre:556638 dre:114457 dre:436768 |
| dre04510 | Focal adhesion | 19/389 | 354/8379 | 0.288228623669938 | 0.816076257715589 | 57934 30429\_2 793158 436718\_1 373107\_1 100000252\_1 386856\_1 563023 novel.946 556842\_1 571431 560564\_2 245702\_1 58100\_1 378480 797491\_1 796537\_1 497384\_1 792699 | actb1 mylpfa cdc42l rhocb birc2 pik3r3b pdgfra flnb - tln2a LOC571431 pdgfc igf1rb badb pdgfaa itgb7 kdrl prkca rac1l | dre:57934 dre:30429 dre:793158 dre:436718 dre:373107 dre:100000252 dre:386856 dre:563023 dre:386856 dre:556842 dre:571431 dre:560564 dre:245702 dre:58100 dre:103909601 dre:797491 dre:796537 dre:497384 dre:792699 |
| dre01240 | Biosynthesis of cofactors | 13/389 | 234/8379 | 0.291640102973138 | 0.816076257715589 | 100536795 403070 406731 100004977\_2 100415796 100007686 570913\_1 407663 64607\_1 334195 445326 767756 678634 | wu:fb14d09 rgn ugt1ab mthfd2 ugt5b5 LOC100007686 ugt5g1 si:ch211-107o10.3 alas2 mat1a akr1a1a ugt5a2 mat2al | dre:100536795 dre:403070 dre:406731 dre:100004977 dre:678597 dre:100007686 dre:570913 dre:407663 dre:64607 dre:334195 dre:445326 dre:767756 dre:678634 |
| dre04070 | Phosphatidylinositol signaling system | 9/389 | 157/8379 | 0.305934815931826 | 0.816076257715589 | 724010 368717\_1 100000252\_1 337236\_1 566642 559938 571356\_1 559198\_1 497384\_1 | dgkaa itpka pik3r3b synj1 tmem55a ppip5k1b pik3c2a mtmr7b prkca | dre:724010 dre:368717 dre:100000252 dre:337236 dre:566642 dre:559938 dre:571356 dre:559198 dre:497384 |
| dre04530 | Tight junction | 16/389 | 300/8379 | 0.318873177286299 | 0.816076257715589 | 337226 57934 286739\_1 569084\_1 793158 436718\_1 81585 792828 60635\_1 100536545 110438642 556298 797651 445305\_1 559392 792699 | zgc:153867 actb1 msna cldni cdc42l rhocb cldnf pard6a cldn7b synpo LOC110438642 micall2b jam3a arpc4l ppp2r2cb rac1l | dre:337226 dre:57934 dre:286739 dre:569084 dre:793158 dre:436718 dre:81585 dre:792828 dre:60635 dre:100536545 dre:110438642 dre:556298 dre:797651 dre:445305 dre:559392 dre:792699 |
| dre00512 | Mucin type O-glycan biosynthesis | 3/389 | 43/8379 | 0.322210683786841 | 0.816076257715589 | 324510 557675 568500 | gcnt4a c1galt1a galnt8a.2 | dre:324510 dre:557675 dre:568500 |
| dre00330 | Arginine and proline metabolism | 5/389 | 81/8379 | 0.323048826511749 | 0.816076257715589 | 100005587\_1 100536795 321892 406688 100006238\_1 | aldh9a1a.1 wu:fb14d09 ckmt1 got2a aldh9a1a.2 | dre:100005587 dre:100536795 dre:321892 dre:406688 dre:100006238 |
| dre00670 | One carbon pool by folate | 2/389 | 25/8379 | 0.324511044849535 | 0.816076257715589 | 100526683\_1 100004977\_2 | aldh1l1 mthfd2 | dre:100526683 dre:100004977 |
| dre03060 | Protein export | 2/389 | 25/8379 | 0.324511044849535 | 0.816076257715589 | 445299 436794\_2 | immp2l sec11a | dre:445299 dre:436794 |
| dre04540 | Gap junction | 9/389 | 160/8379 | 0.325436675373269 | 0.816076257715589 | 386856\_1 406811\_1 novel.946 571431 560564\_2 378480 100000981\_2 566430\_1 497384\_1 | pdgfra zgc:55461 - LOC571431 pdgfc pdgfaa htr2cl1 prkg1b prkca | dre:386856 dre:336681 dre:386856 dre:571431 dre:560564 dre:103909601 dre:100000981 dre:566430 dre:497384 |
| dre00500 | Starch and sucrose metabolism | 3/389 | 44/8379 | 0.335111366763734 | 0.816076257715589 | 406339\_1 100333665\_1 561803 | hk2 gbe1a gyg2 | dre:406339 dre:100333665 dre:561803 |
| dre03460 | Fanconi anemia pathway | 4/389 | 63/8379 | 0.335373804540653 | 0.816076257715589 | 558923\_1 794038\_1 402936 100538153\_1 | palb2 brip1 fancg slx4 | dre:558923 dre:794038 dre:402936 dre:100538153 |
| dre00240 | Pyrimidine metabolism | 5/389 | 84/8379 | 0.350927994855093 | 0.836288790685195 | 492517\_1 100005763\_1 437016\_1 103910413 393744\_2 | zgc:103586 uck2a tk2 tymp dctpp1 | dre:492517 dre:100005763 dre:437016 dre:103910413 dre:393744 |
| dre00630 | Glyoxylate and dicarboxylate metabolism | 3/389 | 46/8379 | 0.360864341186077 | 0.836288790685195 | 322670\_1 541329\_1 792824\_1 | aco2 gcsha pgp | dre:322670 dre:541329 dre:792824 |
| dre03440 | Homologous recombination | 3/389 | 46/8379 | 0.360864341186077 | 0.836288790685195 | 558923\_1 794038\_1 554171 | palb2 brip1 rad52 | dre:558923 dre:794038 dre:554171 |
| dre00020 | Citrate cycle (TCA cycle) | 3/389 | 47/8379 | 0.373689961787099 | 0.843297970984684 | 322670\_1 550579 368262 | aco2 idh3g dlst | dre:322670 dre:550579 dre:368262 |
| dre00400 | Phenylalanine, tyrosine and tryptophan biosynthesis | 1/389 | 10/8379 | 0.378512718177385 | 0.843297970984684 | 406688 | got2a | dre:406688 |
| dre04144 | Endocytosis | 21/389 | 417/8379 | 0.381216890993076 | 0.843297970984684 | 474327\_1 336426\_1 793158 436718\_1 792229 406684 792828 100003902\_1 550542\_1 406837 386856\_1 393881\_1 387529 560210 novel.946 571431 245702\_1 568170\_2 100151129 492487 445305\_1 | capza1b chmp1b cdc42l rhocb sh3glb1a vps26b pard6a si:dkey-16l2.16 chmp4c rnf41 pdgfra vps26a ldlra hsp70l - LOC571431 igf1rb eps15l1a rab11fip4b rab11a arpc4l | dre:474327 dre:336426 dre:793158 dre:436718 dre:792229 dre:406684 dre:792828 dre:100003902 dre:550542 dre:406837 dre:386856 dre:393881 dre:387529 dre:30671 dre:386856 dre:571431 dre:245702 dre:568170 dre:100151129 dre:492487 dre:445305 |
| dre00561 | Glycerolipid metabolism | 5/389 | 89/8379 | 0.397581660697839 | 0.850142330423483 | 724010 100005587\_1 100536795 445326 100006238\_1 | dgkaa aldh9a1a.1 wu:fb14d09 akr1a1a aldh9a1a.2 | dre:724010 dre:100005587 dre:100536795 dre:445326 dre:100006238 |
| dre00600 | Sphingolipid metabolism | 5/389 | 89/8379 | 0.397581660697839 | 0.850142330423483 | 445250 445250\_1 100330155 100000684\_1 553981\_1 | neu3.2 neu3.2 cers4b neu3.5 sptlc1 | dre:445250 dre:445250 dre:100330155 dre:100000684 dre:553981 |
| dre00740 | Riboflavin metabolism | 1/389 | 11/8379 | 0.407400106470968 | 0.850142330423483 | 436725 | acp5b | dre:436725 |
| dre03430 | Mismatch repair | 2/389 | 30/8379 | 0.409102546667238 | 0.850142330423483 | 568323 556995\_2 | mlh3 lig1 | dre:568323 dre:556995 |
| dre00140 | Steroid hormone biosynthesis | 5/389 | 91/8379 | 0.4161851300398 | 0.850142330423483 | 406731 393293 100415796 570913\_1 767756 | ugt1ab hsd11b1la ugt5b5 ugt5g1 ugt5a2 | dre:406731 dre:100006793 dre:678597 dre:570913 dre:767756 |
| dre00120 | Primary bile acid biosynthesis | 2/389 | 31/8379 | 0.425426968767093 | 0.850142330423483 | 723999 795106 | zgc:136333 cyp27a1.2 | dre:795106 dre:795106 |
| dre04623 | Cytosolic DNA-sensing pathway | 4/389 | 72/8379 | 0.431125456348722 | 0.850142330423483 | 140621 394162 140621\_1 561021\_1 | casp3a polr3f casp3a si:dkey-103e21.5 | dre:140621 dre:394162 dre:140621 dre:561021 |
| dre04020 | Calcium signaling pathway | 22/389 | 450/8379 | 0.432260018951145 | 0.850142330423483 | 565241\_2 368717\_1 544664 386856\_1 566671 100005049 novel.946 569566 571431 560564\_2 387296\_1 58147\_1 378480 492486\_1 100000981\_2 353247 259258 796537\_1 100005590\_1 497384\_1 100137117 570493\_1 | ppp3ccb itpka erbb3b pdgfra orai1a fgf1b - LOC569566 LOC571431 pdgfc p2rx1 p2rx3a pdgfaa adra1bb htr2cl1 tnnc1a p2rx4a kdrl hrh2b prkca si:dkey-1h24.2 grin2ab | dre:565241 dre:368717 dre:544664 dre:386856 dre:566671 dre:100005049 dre:386856 dre:569566 dre:571431 dre:560564 dre:387296 dre:58147 dre:103909601 dre:492486 dre:100000981 dre:353247 dre:259258 dre:796537 dre:100005590 dre:497384 dre:100137117 dre:570493 |
| dre04146 | Peroxisome | 6/389 | 114/8379 | 0.436716950560008 | 0.850142330423483 | 450048 406803 562105 100093708 100003383\_1 110440136 | ech1 abcd3a si:dkey-275b16.2 slc25a17 pmvk LOC110440136 | dre:450048 dre:406803 dre:110440136 dre:100093708 dre:100003383 dre:110440136 |
| dre04137 | Mitophagy - animal | 6/389 | 116/8379 | 0.453328310266509 | 0.870867543406715 | 550134 100000242 555713\_1 336159\_1 559564 561933 | ubb zgc:92606 nbr1b optn eif2ak3 rhot1b | dre:550134 dre:100000242 dre:555713 dre:336159 dre:559564 dre:561933 |
| dre03010 | Ribosome | 8/389 | 161/8379 | 0.47389710973696 | 0.898558156124625 | 550564\_1 768187\_1 553284 336712 554098\_2 450009\_1 553799 337007 | faub mrpl18 mrpl1 rpl10 mrpl28 mrps16 mrpl30 rps12 | dre:550564 dre:768187 dre:553284 dre:336712 dre:554098 dre:450009 dre:553799 dre:337007 |
| dre04620 | Toll-like receptor signaling pathway | 6/389 | 121/8379 | 0.494311068697607 | 0.907856532263727 | 100000252\_1 368481 100002946 561021\_1 403131 792699 | pik3r3b stat1b cxcl8a si:dkey-103e21.5 tlr4ba rac1l | dre:100000252 dre:368481 dre:100002946 dre:561021 dre:403131 dre:792699 |
| dre03040 | Spliceosome | 8/389 | 164/8379 | 0.494915427967504 | 0.907856532263727 | 100004402 100005893\_1 566017 100037380\_2 560210 394058\_1 406288\_1 286774\_1 | plrg1 magoh lsm8 zgc:163098 hsp70l fus srsf1a phf5a | dre:100004402 dre:100005893 dre:566017 dre:100037380 dre:30671 dre:394058 dre:406288 dre:286774 |
| dre00250 | Alanine, aspartate and glutamate metabolism | 3/389 | 57/8379 | 0.497455634117111 | 0.907856532263727 | 447815\_1 564754\_1 406688 | rimkla nat8l got2a | dre:447815 dre:564754 dre:406688 |
| dre00562 | Inositol phosphate metabolism | 6/389 | 123/8379 | 0.510424195399933 | 0.91127926365401 | 560753 368717\_1 337236\_1 566479\_1 571356\_1 559198\_1 | tpi1b itpka synj1 plch1 pik3c2a mtmr7b | dre:560753 dre:368717 dre:337236 dre:566479 dre:571356 dre:559198 |
| dre04261 | Adrenergic signaling in cardiomyocytes | 14/389 | 301/8379 | 0.537122074910828 | 0.91127926365401 | 64614 100333318 100037358\_1 793666 559015 678560\_1 562282\_1 555423 492486\_1 353247 550357\_2 114457 559392 497384\_1 | atp1a1a.3 creb3l4 zgc:163073 popdc2 cacnb3b cacnb4b ppp2r5b scn1ba adra1bb tnnc1a cremb atp1b2b ppp2r2cb prkca | dre:64614 dre:100333318 dre:100037358 dre:793666 dre:559015 dre:678560 dre:562282 dre:555423 dre:492486 dre:353247 dre:550357 dre:114457 dre:559392 dre:497384 |
| dre03250 | Viral life cycle - HIV-1 | 4/389 | 83/8379 | 0.542303890267043 | 0.91127926365401 | 559677\_1 321602\_1 562808\_1 559032\_1 | nelfa cdk9 map1sa map1b | dre:559677 dre:321602 dre:562808 dre:559032 |
| dre04916 | Melanogenesis | 8/389 | 171/8379 | 0.542934248059269 | 0.91127926365401 | 100333318 556087\_1 58072 30369\_1 791993 393760\_1 30127\_1 497384\_1 | creb3l4 wnt2bb fzd8b fzd7a wnt4b gnao1a wnt2 prkca | dre:100333318 dre:556087 dre:58072 dre:30369 dre:791993 dre:393760 dre:30127 dre:497384 |
| dre00515 | Mannose type O-glycan biosynthesis | 2/389 | 39/8379 | 0.546558767424871 | 0.91127926365401 | 569769 100093713 | pomt1 zgc:165519 | dre:569769 dre:100093713 |
| dre01040 | Biosynthesis of unsaturated fatty acids | 2/389 | 39/8379 | 0.546558767424871 | 0.91127926365401 | 334217 317738\_1 | elovl7a elovl6 | dre:334217 dre:317738 |
| dre03320 | PPAR signaling pathway | 5/389 | 106/8379 | 0.550483192871752 | 0.91127926365401 | 550134 30355 723999 794841\_1 795106 | ubb apoa1a zgc:136333 plin2 cyp27a1.2 | dre:550134 dre:30355 dre:795106 dre:794841 dre:795106 |
| dre03013 | Nucleocytoplasmic transport | 6/389 | 129/8379 | 0.5575344504303 | 0.91127926365401 | 393693 100005893\_1 407710\_1 324342 100004503\_1 393973\_1 | sap18 magoh xpo7 xpo4 eef1a1b rae1 | dre:393693 dre:100005893 dre:407710 dre:324342 dre:100004503 dre:393973 |
| dre01232 | Nucleotide metabolism | 6/389 | 131/8379 | 0.572768561970119 | 0.91127926365401 | 492517\_1 100005763\_1 437016\_1 103910413 100005067\_1 393744\_2 | zgc:103586 uck2a tk2 tymp dguok dctpp1 | dre:492517 dre:100005763 dre:437016 dre:103910413 dre:100005067 dre:393744 |
| dre04136 | Autophagy - other | 2/389 | 41/8379 | 0.573936177356809 | 0.91127926365401 | 100000242 792689\_1 | zgc:92606 mlst8 | dre:100000242 dre:792689 |
| dre00514 | Other types of O-glycan biosynthesis | 3/389 | 65/8379 | 0.587012637515187 | 0.91127926365401 | 557675 569769 568500 | c1galt1a pomt1 galnt8a.2 | dre:557675 dre:569769 dre:568500 |
| dre03020 | RNA polymerase | 2/389 | 42/8379 | 0.587167346554118 | 0.91127926365401 | 394162 325603 | polr3f polr1b | dre:394162 dre:325603 |
| dre04625 | C-type lectin receptor signaling pathway | 7/389 | 156/8379 | 0.591647667068199 | 0.91127926365401 | 436718\_1 565241\_2 100000252\_1 795414\_2 571242\_2 368481 561021\_1 | rhocb ppp3ccb pik3r3b relb malt3 stat1b si:dkey-103e21.5 | dre:436718 dre:565241 dre:100000252 dre:795414 dre:571242 dre:368481 dre:561021 |
| dre00604 | Glycosphingolipid biosynthesis - ganglio series | 1/389 | 19/8379 | 0.595139854301192 | 0.91127926365401 | 394230\_1 | st3gal5 | dre:394230 |
| dre00565 | Ether lipid metabolism | 3/389 | 67/8379 | 0.607750176418575 | 0.91127926365401 | 406370\_1 767688 100148818 | pla2g6 zgc:154040 si:ch73-55i23.1 | dre:406370 dre:767688 dre:100148818 |
| dre00603 | Glycosphingolipid biosynthesis - globo and isoglobo series | 1/389 | 20/8379 | 0.613978442420431 | 0.91127926365401 | 100093713 | zgc:165519 | dre:100093713 |
| dre03420 | Nucleotide excision repair | 3/389 | 68/8379 | 0.617856179875265 | 0.91127926365401 | 393774\_2 556995\_2 553405\_1 | pole3 lig1 pole | dre:393774 dre:556995 dre:553405 |
| dre03022 | Basal transcription factors | 2/389 | 45/8379 | 0.62502226976745 | 0.91127926365401 | 798997\_2 567899 | gtf2a2 gtf2e1 | dre:798997 dre:567899 |
| dre04330 | Notch signaling pathway | 4/389 | 93/8379 | 0.632905649072075 | 0.91127926365401 | 678649 393500 557340 140422\_1 | zgc:136929 zgc:66440 atxn1a jag2b | dre:678649 dre:393500 dre:557340 dre:140422 |
| dre00062 | Fatty acid elongation | 2/389 | 46/8379 | 0.637029448339331 | 0.91127926365401 | 334217 317738\_1 | elovl7a elovl6 | dre:334217 dre:317738 |
| dre00280 | Valine, leucine and isoleucine degradation | 3/389 | 71/8379 | 0.647105491630857 | 0.91127926365401 | 100005587\_1 100536795 100006238\_1 | aldh9a1a.1 wu:fb14d09 aldh9a1a.2 | dre:100005587 dre:100536795 dre:100006238 |
| dre00520 | Amino sugar and nucleotide sugar metabolism | 3/389 | 74/8379 | 0.674735145683745 | 0.91127926365401 | 406339\_1 100007686 563138 | hk2 LOC100007686 si:ch211-219a15.4 | dre:406339 dre:100007686 dre:563138 |
| dre00100 | Steroid biosynthesis | 1/389 | 24/8379 | 0.680984103765636 | 0.91127926365401 | 494054\_1 | tm7sf2 | dre:494054 |
| dre00910 | Nitrogen metabolism | 1/389 | 24/8379 | 0.680984103765636 | 0.91127926365401 | 100006448 | ca6 | dre:100006448 |
| dre04520 | Adherens junction | 8/389 | 194/8379 | 0.685201492335713 | 0.91127926365401 | 57934 30429\_2 793158 436718\_1 569566 571431 245702\_1 792699 | actb1 mylpfa cdc42l rhocb LOC569566 LOC571431 igf1rb rac1l | dre:57934 dre:30429 dre:793158 dre:436718 dre:569566 dre:571431 dre:245702 dre:792699 |
| dre04140 | Autophagy - animal | 10/389 | 241/8379 | 0.688403162830248 | 0.91127926365401 | 321622 100000242 792229 100000252\_1 573492 792689\_1 245702\_1 58100\_1 559564 563140\_1 | hmgb1a zgc:92606 sh3glb1a pik3r3b rragca mlst8 igf1rb badb eif2ak3 si:ch211-11n16.2 | dre:110438871 dre:100000242 dre:792229 dre:100000252 dre:573492 dre:792689 dre:245702 dre:58100 dre:559564 dre:563140 |
| dre04141 | Protein processing in endoplasmic reticulum | 10/389 | 241/8379 | 0.688403162830248 | 0.91127926365401 | 327197 386590 266757\_1 323883 565338\_1 560210 564295\_2 100005358 559564 393877 | vcp hsp90b1 tram1 cul1a eif2ak1 hsp70l traf2a si:ch211-274f20.2 eif2ak3 derl3 | dre:327197 dre:386590 dre:266757 dre:323883 dre:565338 dre:30671 dre:564295 dre:100005358 dre:559564 dre:393877 |
| dre04622 | RIG-I-like receptor signaling pathway | 3/389 | 76/8379 | 0.692252558801204 | 0.91127926365401 | 564295\_2 100002946 561021\_1 | traf2a cxcl8a si:dkey-103e21.5 | dre:564295 dre:100002946 dre:561021 |
| dre00970 | Aminoacyl-tRNA biosynthesis | 2/389 | 51/8379 | 0.692567742170248 | 0.91127926365401 | 324940\_1 394188 | aars qars | dre:792787 dre:394188 |
| dre01250 | Biosynthesis of nucleotide sugars | 2/389 | 51/8379 | 0.692567742170248 | 0.91127926365401 | 406339\_1 100007686 | hk2 LOC100007686 | dre:406339 dre:100007686 |
| dre00563 | Glycosylphosphatidylinositol (GPI)-anchor biosynthesis | 1/389 | 25/8379 | 0.69583714788714 | 0.91127926365401 | 561471 | pigl | dre:561471 |
| dre04012 | ErbB signaling pathway | 5/389 | 127/8379 | 0.709022244610687 | 0.91127926365401 | 100000252\_1 544664 571431 58100\_1 497384\_1 | pik3r3b erbb3b LOC571431 badb prkca | dre:100000252 dre:544664 dre:571431 dre:58100 dre:497384 |
| dre04340 | Hedgehog signaling pathway | 3/389 | 79/8379 | 0.7171840303151 | 0.91127926365401 | 323883 30269\_1 100006858\_1 | cul1a shha csnk1e | dre:323883 dre:30269 dre:100006858 |
| dre00531 | Glycosaminoglycan degradation | 1/389 | 27/8379 | 0.723505656450873 | 0.91127926365401 | 565246 | si:dkey-192p21.6 | dre:565246 |
| dre04270 | Vascular smooth muscle contraction | 9/389 | 226/8379 | 0.729228943560355 | 0.91127926365401 | 337226 436718\_1 406370\_1 436966 403114 492486\_1 566430\_1 100148818 497384\_1 | zgc:153867 rhocb pla2g6 zgc:91818 adm2a adra1bb prkg1b si:ch73-55i23.1 prkca | dre:337226 dre:436718 dre:406370 dre:436966 dre:403114 dre:492486 dre:566430 dre:100148818 dre:497384 |
| dre00061 | Fatty acid biosynthesis | 1/389 | 28/8379 | 0.736383565890601 | 0.91127926365401 | 559001\_1 | fasn | dre:559001 |
| dre00770 | Pantothenate and CoA biosynthesis | 1/389 | 28/8379 | 0.736383565890601 | 0.91127926365401 | 100536795 | wu:fb14d09 | dre:100536795 |
| dre04514 | Cell adhesion molecules | 8/389 | 204/8379 | 0.737610535404878 | 0.91127926365401 | 569084\_1 81585 60635\_1 557935 110438642 797651 101886406 797491\_1 | cldni cldnf cldn7b zmp:0000001082 LOC110438642 jam3a cd28 itgb7 | dre:569084 dre:81585 dre:60635 dre:557935 dre:110438642 dre:797651 dre:101886406 dre:797491 |
| dre04110 | Cell cycle | 9/389 | 229/8379 | 0.743312807926212 | 0.91127926365401 | 431763\_1 323299\_2 323883 novel.902 797406\_2 563708 324475\_1 562282\_1 393223\_1 | gadd45ab atrx cul1a - espl1 skp2 smc3 ppp2r5b fzr1a | dre:431763 dre:323299 dre:323883 dre:323299 dre:797406 dre:563708 dre:324475 dre:562282 dre:393223 |
| dre04912 | GnRH signaling pathway | 6/389 | 158/8379 | 0.74899665505809 | 0.91127926365401 | 793158 566945 30498 571431 100148818 497384\_1 | cdc42l mmp14b egr1 LOC571431 si:ch73-55i23.1 prkca | dre:793158 dre:566945 dre:30498 dre:571431 dre:100148818 dre:497384 |
| dre04145 | Phagosome | 8/389 | 209/8379 | 0.761460505155385 | 0.916737524787254 | 57934 359840\_1 406811\_1 571584 100005358 403131 792699 100007982 | actb1 atp6v1b2 zgc:55461 marco si:ch211-274f20.2 tlr4ba rac1l hbl1 | dre:57934 dre:359840 dre:336681 dre:571584 dre:100005358 dre:403131 dre:792699 dre:100007982 |
| dre04068 | FoxO signaling pathway | 8/389 | 210/8379 | 0.766040945370171 | 0.916737524787254 | 324140 100000242 431763\_1 100000252\_1 571431 245702\_1 563708 100006858\_1 | sgk1 zgc:92606 gadd45ab pik3r3b LOC571431 igf1rb skp2 csnk1e | dre:324140 dre:100000242 dre:431763 dre:100000252 dre:571431 dre:245702 dre:563708 dre:100006858 |
| dre02010 | ABC transporters | 2/389 | 63/8379 | 0.797588206992854 | 0.943628119121661 | 406803 553281 | abcd3a abcc8 | dre:406803 dre:553281 |
| dre01212 | Fatty acid metabolism | 3/389 | 91/8379 | 0.801437580623876 | 0.943628119121661 | 334217 559001\_1 317738\_1 | elovl7a fasn elovl6 | dre:334217 dre:559001 dre:317738 |
| dre00220 | Arginine biosynthesis | 1/389 | 35/8379 | 0.811242193218555 | 0.947530881679272 | 406688 | got2a | dre:406688 |
| dre04114 | Oocyte meiosis | 6/389 | 182/8379 | 0.855665686804762 | 0.985912062293762 | 565241\_2 323883 797406\_2 245702\_1 324475\_1 562282\_1 | ppp3ccb cul1a espl1 igf1rb smc3 ppp2r5b | dre:565241 dre:323883 dre:797406 dre:245702 dre:324475 dre:562282 |
| dre04218 | Cellular senescence | 8/389 | 234/8379 | 0.857608437748684 | 0.985912062293762 | 565241\_2 431763\_1 100000252\_1 393176\_1 557551 100037341 100002946 572042 | ppp3ccb gadd45ab pik3r3b rad9a hipk2 zgc:162895 cxcl8a hipk1a | dre:565241 dre:431763 dre:100000252 dre:393176 dre:557551 dre:100037341 dre:100002946 dre:572042 |
| dre00601 | Glycosphingolipid biosynthesis - lacto and neolacto series | 1/389 | 42/8379 | 0.864882168969353 | 0.986506223980668 | 100093713 | zgc:165519 | dre:100093713 |
| dre04142 | Lysosome | 7/389 | 214/8379 | 0.874928795175501 | 0.987305045903365 | 571066 406511 553423\_1 337771 436725 565246 445244\_1 | si:ch1073-513e17.1 sort1a sumf1 zgc:162858 acp5b si:dkey-192p21.6 ap1m3 | dre:571066 dre:406511 dre:553423 dre:337771 dre:436725 dre:565246 dre:445244 |
| dre00564 | Glycerophospholipid metabolism | 4/389 | 135/8379 | 0.879107232653681 | 0.987305045903365 | 724010 406370\_1 767688 100148818 | dgkaa pla2g6 zgc:154040 si:ch73-55i23.1 | dre:724010 dre:406370 dre:767688 dre:100148818 |
| dre03008 | Ribosome biogenesis in eukaryotes | 2/389 | 83/8379 | 0.903641336965326 | 0.990867985965188 | 393107 407084 | utp6 riok2 | dre:393107 dre:407084 |
| dre00190 | Oxidative phosphorylation | 5/389 | 171/8379 | 0.904580963739535 | 0.990867985965188 | 393793\_1 359840\_1 447942\_1 796997\_1 436768 | uqcrc1 atp6v1b2 cox6a2 ndufs7 uqcrq | dre:393793 dre:359840 dre:447942 dre:796997 dre:436768 |
| dre04814 | Motor proteins | 10/389 | 305/8379 | 0.907500902726723 | 0.990867985965188 | 474327\_1 337226 57934 30429\_2 334274 100149148 406811\_1 100037358\_1 353247 556023 | capza1b zgc:153867 actb1 mylpfa myhc4 myha zgc:55461 zgc:163073 tnnc1a dnah2 | dre:474327 dre:337226 dre:57934 dre:30429 dre:334274 dre:100149148 dre:336681 dre:100037358 dre:353247 dre:556023 |
| dre00480 | Glutathione metabolism | 2/389 | 85/8379 | 0.910754040638858 | 0.990867985965188 | 553575\_1 406703 | gsr gsta.1 | dre:553575 dre:406703 |
| dre04130 | SNARE interactions in vesicular transport | 1/389 | 56/8379 | 0.930824358557943 | 0.990867985965188 | 393294 | use1 | dre:393294 |
| dre04910 | Insulin signaling pathway | 6/389 | 212/8379 | 0.933755696675258 | 0.990867985965188 | 100001260 100000252\_1 79380\_1 406339\_1 58100\_1 559001\_1 | ppp1r3ab pik3r3b eif4ea hk2 badb fasn | dre:100001260 dre:100000252 dre:79380 dre:406339 dre:58100 dre:559001 |
| dre04744 | Phototransduction | 1/389 | 57/8379 | 0.934057486579204 | 0.990867985965188 | 100330921 | si:ch211-103a14.5 | dre:100330921 |
| dre04914 | Progesterone-mediated oocyte maturation | 3/389 | 133/8379 | 0.950486230952899 | 0.990867985965188 | 100000252\_1 245702\_1 393223\_1 | pik3r3b igf1rb fzr1a | dre:100000252 dre:245702 dre:393223 |
| dre03050 | Proteasome | 1/389 | 65/8379 | 0.955045647865661 | 0.990867985965188 | 796450 | adrm1 | dre:796450 |
| dre04371 | Apelin signaling pathway | 6/389 | 226/8379 | 0.955219671657079 | 0.990867985965188 | 30498 100331581\_1 335655 100037358\_1 321449\_1 557486 | egr1 si:dkey-44g17.6 gngt2a zgc:163073 ctgfa hdac5 | dre:30498 dre:100331581 dre:335655 dre:100037358 dre:321449 dre:557486 |
| dre00510 | N-Glycan biosynthesis | 1/389 | 68/8379 | 0.961065751037839 | 0.990867985965188 | 327601\_1 | alg8 | dre:327601 |
| dre04920 | Adipocytokine signaling pathway | 2/389 | 108/8379 | 0.963946155495466 | 0.990867985965188 | 100037357 564295\_2 | tnfrsf1b traf2a | dre:100037357 dre:564295 |
| dre04060 | Cytokine-cytokine receptor interaction | 6/389 | 242/8379 | 0.971918931166661 | 0.990867985965188 | 100037357 799354\_1 368326 100006078\_1 562247\_1 100002946 | tnfrsf1b cxcl12b il13ra2 ccr9b clcf1 cxcl8a | dre:100037357 dre:799354 dre:368326 dre:100006078 dre:562247 dre:100002946 |
| dre04350 | TGF-beta signaling pathway | 3/389 | 161/8379 | 0.982102986183638 | 0.990867985965188 | 436718\_1 323883 556634 | rhocb cul1a adamtsl7 | dre:436718 dre:323883 dre:556634 |
| dre04512 | ECM-receptor interaction | 2/389 | 128/8379 | 0.984081218938029 | 0.990867985965188 | 563428 797491\_1 | fras1 itgb7 | dre:563428 dre:797491 |
| dre00230 | Purine metabolism | 2/389 | 192/8379 | 0.998968610817199 | 0.998968610817199 | 777719 100005067\_1 | papss2a dguok | dre:777719 dre:100005067 |
